# Supplementary material for: Causal Relationship between Aging and Anorexia Nervosa: A White-Matter-Microstructure-Mediated Mendelian Randomization Analysis
Source: Biomedicines. 2024 Aug 16;12(8):1874. doi: 10.3390/biomedicines12081874 (PMC11351342; doi:10.3390/biomedicines12081874)
Supplement: Supplementary file 1 [file biomedicines-12-01874-s001.zip › Supplementary Figure/Figure S8/Leaveoneout plot of MR analysis for TL to ubm-b-1468.pdf]

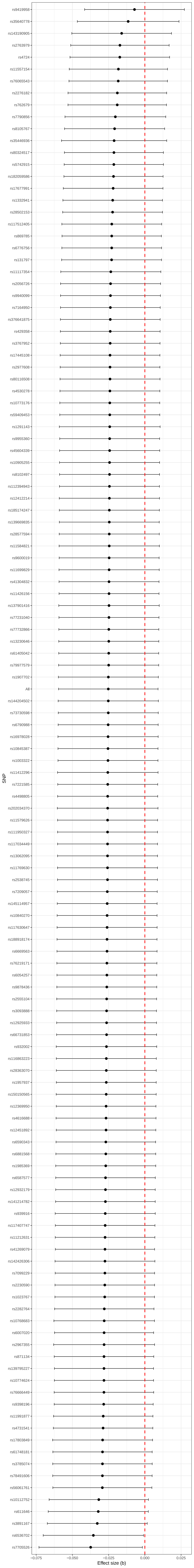

rs9419958
rs35640778
rs143190905
rs2763979
rs4724
rs11557154
rs76065543
rs2276182
rs762679
rs7790856
rs8105767
rs35446936
rs80324517
rs5742915
rs182059586
rs17677991
rs1332941
rs28502153
rs117512405
rs869785
rs6776756
rs131797
rs11117354
rs2056726
rs9940099
rs7164950
rs376641875
rs429358
rs3767952
rs17445108
rs2977608
rs80116508
rs4530278
rs10773176
rs59409453
rs1291143
rs9955360
rs45604339
rs10905255
rs8102497
rs112394943
rs12412214
rs185174247
rs139669835
rs28577594
rs11584821
rs9600019
rs11699829
rs41304832
rs11426156
rs137901416
rs77231040
rs77732866
rs13230646
rs61405042
rs79977579
rs1907702
A8
rs144204502
rs73730598
rs6790988
rs16978028
rs10845387
rs1003322
rs11412296
rs7221585
rs4498805
rs202034370
rs11579626
rs111950327
rs117034449
rs13062095
rs11769630
rs2538745
rs7209057
rs145114957
rs10840270
rs117630647
rs188918174
rs6669563
rs76219171
rs6054257
rs9878436
rs2555104
rs3093888
rs12925933
rs66731853
rs932002
rs116863223
rs28363070
rs1957937
rs150150565
rs12369950
rs4616688
rs12451892
rs6590343
rs6881568
rs1985369
rs6587577
rs12932179
rs141214782
rs939916
rs117407747
rs11212631
rs41269079
rs142426306
rs7099229
rs2230590
rs1023767
rs2282764
rs10768683
rs6007020
rs2967355
rs871134
rs139795227
rs10774624
rs76666449
rs9398196
rs11991877
rs4731541
rs17803849
rs61748181
rs3785074
rs78491606
rs56061761
rs10112752
rs611646
rs3891167
rs6536702
rs7705526
SNP
-0.075
-0.050
-0.025
0.000
0.025
Effect size (b)
